# Visualization of Sialidase Activity in Mammalian Tissues and Cancer Detection with a Novel Fluorescent Sialidase Substrate

**DOI:** 10.1371/journal.pone.0081941

**Published:** 2014-01-10

**Authors:** Akira Minami, Tadamune Otsubo, Daisuke Ieno, Kiyoshi Ikeda, Hiroaki Kanazawa, Kosuke Shimizu, Ko Ohata, Tsunehiro Yokochi, Yuuki Horii, Hokuto Fukumoto, Risa Taguchi, Tadanobu Takahashi, Naoto Oku, Takashi Suzuki

**Affiliations:** 1 Department of Biochemistry, School of Pharmaceutical Sciences, University of Shizuoka, Shizuoka, Japan; 2 Department of Organic Chemistry, School of Pharmaceutical Sciences, Hiroshima International University, Hiroshima, Japan; 3 Department of Functional Anatomy, School of Nursing, University of Shizuoka, Shizuoka, Japan; 4 Department of Medical Biochemistry, School of Pharmaceutical Sciences, University of Shizuoka, Shizuoka, Japan; 5 Department of Gastroenterological Surgery, Shizuoka General Hospital, Shizuoka, Japan; 6 Department of Medical Technology, Shizuoka General Hospital, Shizuoka, Japan; University of Insubria, Italy

## Abstract

Sialidase removes sialic acid from sialoglycoconjugates and plays crucial roles in many physiological and pathological processes. Various human cancers express an abnormally high level of the plasma membrane-associated sialidase isoform.Visualization of sialidase activity in living mammalian tissues would be useful not only for understanding sialidase functions but also for cancer diagnosis. However, since enzyme activity of mammalian sialidase is remarkably weak compared with that of bacterial and viral sialidases, it has been difficult to detect sialidase activity in mammalian tissues. We synthesized a novel benzothiazolylphenol-based sialic acid derivative (BTP-Neu5Ac) as a fluorescent sialidase substrate. BTP-Neu5Ac can visualize sialidase activities sensitively and selectively in acute rat brain slices. Cancer cells implanted orthotopically in mouse colons and human colon cancers (stages T3-T4) were also clearly detected with BTP-Neu5Ac. The results suggest that BTP-Neu5Ac is useful for histochemical imaging of sialidase activities.

## Introduction

Sialidase (EC 3.2.1.18) removes sialic acid from sialoglycoconjugates, such as glycoproteins and glycolipids. Mammalian sialidase is known to have 4 isoforms (NEU1, NEU2, NEU3 and NEU4) and plays many roles in cell functions including differentiation, growth, apoptosis and migration and in survival and proliferation of cancer cells [1], [2]. Visualizing the detailed distribution of sialidase activity in mammalian tissue can help us to understand the physiological and pathological roles of sialidase. Additionally, since the expression level of NEU3, a plasma membrane-associated sialidase, is remarkably increased in various human cancers such as colon, renal, prostate and ovarian cancers [1], [2], [3], detection of membrane sialidase activities in viable cancer tissue will also be useful for cancer diagnosis and real-time monitoring of cancer during a surgical operation.

X-Neu5Ac (5-bromo-4-chloroindol-3-yl-α-D-N-acetylneuraminic acid) is a widely used artificial sialidase substrate for cytochemical and histochemical imaging of sialidase activity. Compound X (5-bromo-4-chloro-3-hydroxyindole) is released from X-Neu5Ac with sialidase and oxidized to a water-insoluble visible indigo blue. To enhance the specificity of staining, indigogenic substrates are often used with an equimolar mixture of K_3_[Fe(CN)_6_] and K_4_[Fe(CN)_6_] as an oxidation catalyst. However, the sensitivity is not sufficient to observe the detailed distribution of sialidase activity in mammalian tissues [4]. Enzyme activity of mammalian sialidase is remarkably low compared to that of bacteria and virus [5], [6]. To enhance the sensitivity of X-Neu5Ac, a sensitizer such as Fast Red Violet LB (FRV LB) as a coupler to form an azo dye is used with X-Neu5Ac [6], [7], [8]. However, since a two-step reaction is needed for staining with the FRV LB, nonspecific staining caused by the sensitizer is unavoidable and makes it difficult to use, especially in clinical fields. In the present study, we developed novel fluorescent sialidase substrates, benzothiazolylphenol-based sialic acid derivatives (BTP-Neu5Ac), for highly sensitive and specific visualization of sialidase activity in living mammalian tissues by a single-step reaction.

In the present study, we found that BTP-Neu5Ac can visualize sialidase activities sensitively and selectively in acute rat brain slices. BTP-Neu5Ac can also clearly detect cancer cells implanted orthotopically in mouse colons and human colon cancers.

## Materials and Methods

### Synthetic procedures

The synthesis of compounds is described in detail in File S1.

### Materials

The following products were purchased from the vendors indicated: sialidase from *Arthrobacter ureafaciens* (AUSA, recombinant expressed in *Escherichia. coli*, Calbiochem, San Diego, CA, USA), X-Neu5Ac (Peptide Institute, Osaka, Japan), halothane (Takeda Pharmaceutical Company, Osaka, Japan), Dulbecco's modified Eagle's medium (DMEM, Wako Pure Chemical Industries, Osaka, Japan), penicillin and streptomycin (MP Biomedicals, Santa Ana, CA, USA), fetal bovine serum (FBS, AusGeneX, Molendinar, QLD, Australia), phycoerythrin (PE)-conjugated goat anti-rabbit IgG (Santa Cruz Biotechnology, Dallas, TX, USA) and rabbit anti-CD71 (transferrin receptor) polyclonal antibody (Assay Biotechnology, Sunnyvale, CA, USA). Reagents used for making buffer solutions were purchased from Wako Pure Chemical Industries.

### Experimental animals

Rats and mice were purchased from Japan SLC (Hamamatsu, Japan). They were housed under standard laboratory conditions (23±1°C, 55±5% humidity) and had access to tap water and diet *ad libitum*. The lights were automatically turned on at 8:00 and turned off at 20:00. All experiments were performed in accordance with the Japanese Pharmacological Society guide for the care and use of laboratory animals, and the protocols were pre-approved by the Animal Ethical Committee of the University of Shizuoka.

### Spectrum analysis

Fluorescent spectra of 5 mM BTP2, BTP3 and BTP4 in artificial cerebrospinal fluid (ACSF, pH 7.3, 200 µl) containing 119 mM NaCl, 2.5 mM KCl, 2.5 mM CaCl_2_, 1.3 mM MgCl_2_, 1.0 mM NaH_2_PO_4_, 26.2 mM NaHCO_3_ and 11 mM D-glucose were measured with a microplate reader, Infinite M200 (Tecan, Männedorf, Schweiz). Excitation spectra were acquired with emission wavelengths at 518 nm for BTP2, 526 nm for BTP3 and 542 nm for BTP4. Emission spectra were acquired with excitation wavelengths at 370 nm for BTP2, 372 nm for BTP3 and 374 nm for BTP4. Fluorescence views of 5 mM BTP2,BTP3 and BTP4 in ACSF and BTP2, BTP3 and BTP4 on a filter paper were imaged under excitation UV light of 365 nm (Vilber Lourmat, Marne-la-Vallée, France) with a digital camera, CX1 (Ricoh, Tokyo, Japan).

### Quantitative analysis of sialidase activity

AUSA (10^0^–10^5.7^ µU ml^−1^: one unit was defined as the amount of enzyme that catalyzed the release of 1 µmol of sialic acid for 1 min.) was incubated in 100 µl of 100 mM sodium phosphate buffer (pH 7.3) containing 10 µM BTP2-Neu5Ac, BTP3-Neu5Ac, BTP4-Neu5Ac or resorufin-Neu5Ac (Res-Neu5Ac) at 37°C for 60 min in a 96-well black microplate (Corning, Corning, NY, USA). The reaction was terminated by addition of 150 µl of sodium carbonate (500 mM, pH 10.7) to each well. Intensity of fluorescence was measured with a microplate reader (ex/em: 370 nm/518 nm for BTP2, 372 nm/526 nm for BTP3, 374 nm/542 nm for BTP4 and 570 nm/585 nm for resorufin).

### Detection of sialidase activity on a PVDF membrane

AUSA (10^−1^–10^4^ µU) was blotted on a polyvinylidine difluoride (PVDF) membrane by using a 96-well dotblotter (circle well size: 28.3 mm^2^, Sanplatec, Osaka, Japan). Each well was washed with 100 mM sodium phosphate buffer (pH 7.3), and then 50 µl of 10 µM BTP4-Neu5Ac or X-Neu5Ac in sodium phosphate buffer was added. After incubation at 37°C for 6 hr, reaction solutions were removed, and then the PVDF membranes were observed with a digital camera under UV light (365 nm) for BTP4-Neu5Ac or with a stereo microscope SZX7 (Olympus, Tokyo, Japan) under visible light for X-Neu5Ac.

### Imaging of sialidase activity in brain slices

Wistar rats (male, 7–10 weeks old, n = 3) were anesthetized with halothane and decapitated. The brain of each rat was quickly harvested and immersed in ice-cold ACSF to suppress excessive neuronal excitation and damage. ACSF used in this experiment was continuously bubbled with 95% O_2_ and 5% CO_2_. Coronal brain slices (400 µm in thickness) were prepared by using a LinearSlicer PRO-7 (Dosaka EM, Kyoto, Japan) in ice-cold ACSF. Acute brain slices were maintained in ACSF at room temperature for at least 60 min and then incubated with 400 µl ACSF containing 1 mM BTP2-Neu5Ac (n = 3), BTP3-Neu5Ac (n = 4) or BTP4-Neu5Ac (n = 4) at 27°C for 1 hr. Incubation chambers were continuously bubbled with 95% O_2_ and 5% CO_2_ during staining. Slices were washed three times with ACSF and transferred to IWAKI 3.5 mm glass-based dishes (Asahi Glass, Tokyo, Japan) filled with ACSF. Fluorescence was observed by using an IX71 fluorescence microscope (excitation filter/emission filter: BP330-385/BA420 or BP330-385/BA510IF, Olympus). Background level of fluorescence for sialidase activity imaging was determined by incubating the brain slices in ACSF without a substrate. In all observations with the fluorescence microscope, gain of a DP70 Digital Microscope Camera (Olympus) was set not to detect background fluorescence.

For whole area imaging of the brain slice, pictures were taken sequentially and each piece was tiled using Photoshop CS4 (Adobe Systems, San Jose, CA, USA). When needed, same processing was performed in the following experiment.

### Cytotoxicity

MDCK cells were exposed to a serum-free medium (SFM) containing 10 or 100 µM BTP-Neu5Ac or BTP. Released LDH was measured using a coupled enzymatic assay (CytoTox-OneTM, Promega, WI, USA).

### Mouse colon tumor model

Murine Colon 26 NL-17 carcinoma cells were cultured in DMEM supplemented with 10% FBS, 100 units mL^−1^ penicillin and 100 µg mL^−1^ streptomycin at 37°C in the presence of 5% CO_2_ in a humid atmosphere. Colon cancer cells were implanted orthotopically following a protocol based on the method reported by Takahashi *et al.* [9] BALB/cCrSlc mice (male, 6 weeks old) were fasted for 12 hr and then anesthetized with chloral hydrate (400 mg kg^−1^ body weight). To induce colitis, the mice were injected intrarectally with 200 µl of 0.1 M HCl into a colon site 1.5 cm distant from the anus via the anus. After 10 min, the mice were injected intrarectally with 200 µl of 0.1 M KOH for neutralization, followed by injection with 400 µl of PBS. After 9 hr, the mice were anesthetized again and injected intrarectally with 200 µl of Colon26 NL-17 cells (8×10^6^ cells) suspended with serum-free DMEM in the same manner. The anus was immediately clamped with a small klemme for 2 hr. At 1 (n = 3) or 2 weeks (n = 3) after orthotopic implantation of colon cancer, colons were harvested after intracardiac perfusion with PBS to remove blood from the whole body.

### Human colon cancer specimens

Two surgical specimens were obtained from human colon adenocarcinoma (UICC T classification T3 and T4) and stained with BTP4-Neu5Ac within 2 hr after surgery. The study was approved by the Ethics Committee of Shizuoka General Hospital (Protocol 13-03-59) and University of Shizuoka (Protocol 25-2) and is in line with the Declaration of Human Rights, Helsinki, 2002. All patients gave written informed consent.

### Detection of colon cancer with BTP4-Neu5Ac

Mouse and human colon cancer tissues were incubated in PBS containing 100–200 µM BTP4-Neu5Ac at 37°C for 60 min. After washing with PBS, fluorescence was observed with a fluorescence microscope (excitation filter/emission filter: BP330-385/BA420) or IVIS Imaging System (PerkinElmer, Waltham, MA, USA). Background level of fluorescence was determined by using normal colons incubated in PBS without BTP4-Neu5Ac.

### Histopathological stains

Mouse and human colons that had been used for sialidase activity imaging were fixed with 4% paraformaldehyde. After being embedded with paraffin, the tissue was cut into 4–7-µm-thick sections and stained by using rabbit anti-CD71 antibody as the primary antibody, PE-conjugated goat anti-rabbit IgG antibody as the secondary antibody and 4′,6-diamidino-2-phenylindole (DAPI, Dojindo Laboratories, Kumamoto, Japan).

### Reproducibility

All stainings were repeated at least twice and reproducibility was confirmed.

## Results and Discussion

### Design of novel sialidase substrate for histochemical imaging

**Figure 1:**
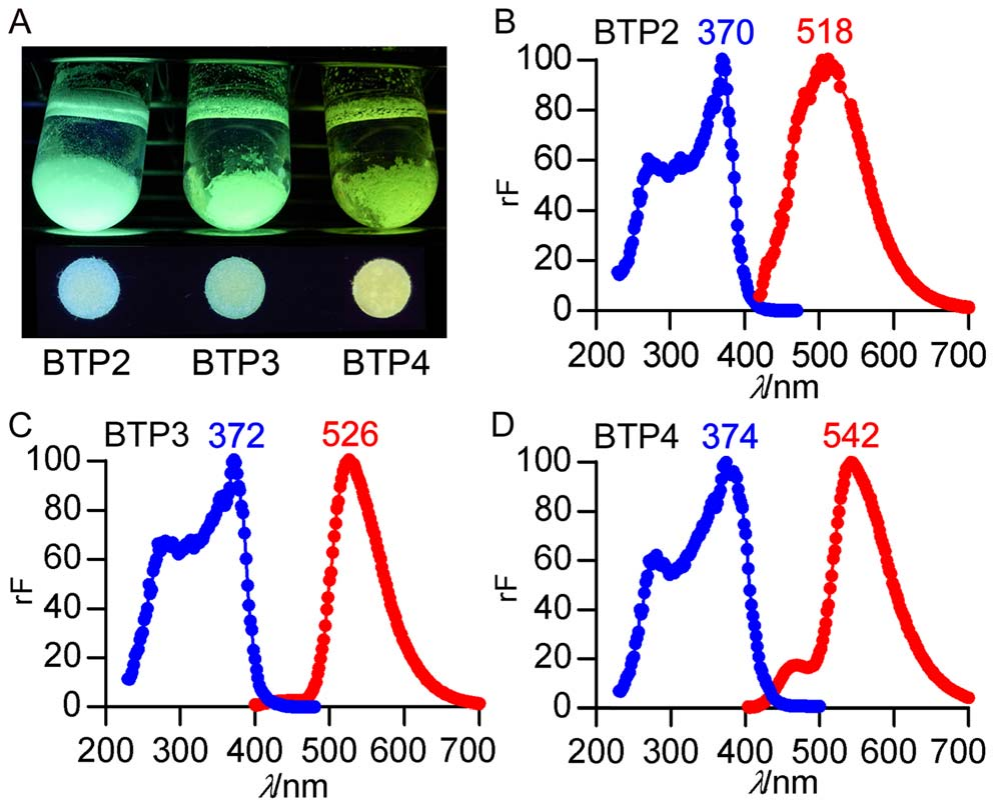
BTP derivatives having different excitation and emission spectra. A, Fluorescence views of BTP2, BTP3 and BTP4 in ACSF (upper) and on filter papers under 365 nm UV light (lower) are shown. B–D, Excitation (blue dots and lines) and emission (red dots and lines) spectra of BTP2 (B), BTP3 (C) and BTP4 (D) were measured in ACSF (pH 7.3). Blue and red numbers represent wave length (nm) at peak fluorescence intensity. Abbreviations: rF, relative fluorescence intensity.

To visualize sialidase activity on the tissue, the aglycone of the sialidase substrate should be a water-insoluble staining dye to be attached to the tissue. Since the fluorophore of 4-methylumbelliferyl-α-D-*N*-acetylneuraminic acid (4MU-Neu5Ac), a commonly used artificial sialidase substrate for quantification of enzyme activity, is water-soluble, 4MU-Neu5Ac is not suitable for histochemical imaging of sialidase activity. Benzothiazolylphenol (BTP), a water-insoluble fluorophore, shows intense fluorescence in a solid condition [10]. BTP-based fluorescent probes have been used for some biological and chemical indicators [11], [12], [13]. When BTP derivatives, BTP2 [14], BTP3 [15] and BTP4 [15], were put in an artificial cerebrospinal fluid (ACSF, pH 7.3), they were precipitated on the bottom of test tubes (Figure 1 A).

BTP2, BTP3 and BTP4 have different emission and excitation spectra (Figure 1 B–D). Since fluorescent staining dyes having different excitation and emission spectra have an advantage over selection of filter sets to take fluorescence images, we used these three BTP derivatives as the aglycone of the sialidase substrate.

### Hydrolysis of BTP-Neu5Ac with sialidase from *Arthrobacter ureafaciens*

**Figure 2:**
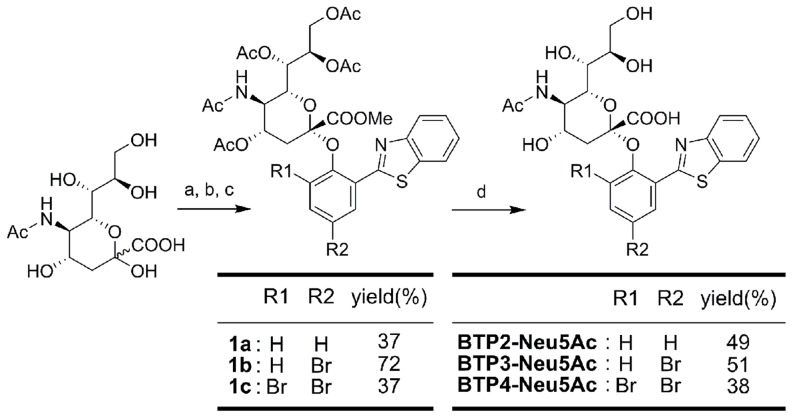
Synthesis of BTP2-Neu5Ac, BTP3-Neu5Ac and BTP4-Neu5Ac. Conditions: (a) Amberlite IR-120(H^+^), dry MeOH, overnight, 92% yield. (b) AcCl-AcOH, overnight, quant. (c) BTP2∼4, NaH, THF-DMF, room temperature, overnight. (d) NaOMe, dry MeOH, 6 hr, room temperature, then NaOH aq., MeOH, room temperature, 2 days.

**Figure 3:**
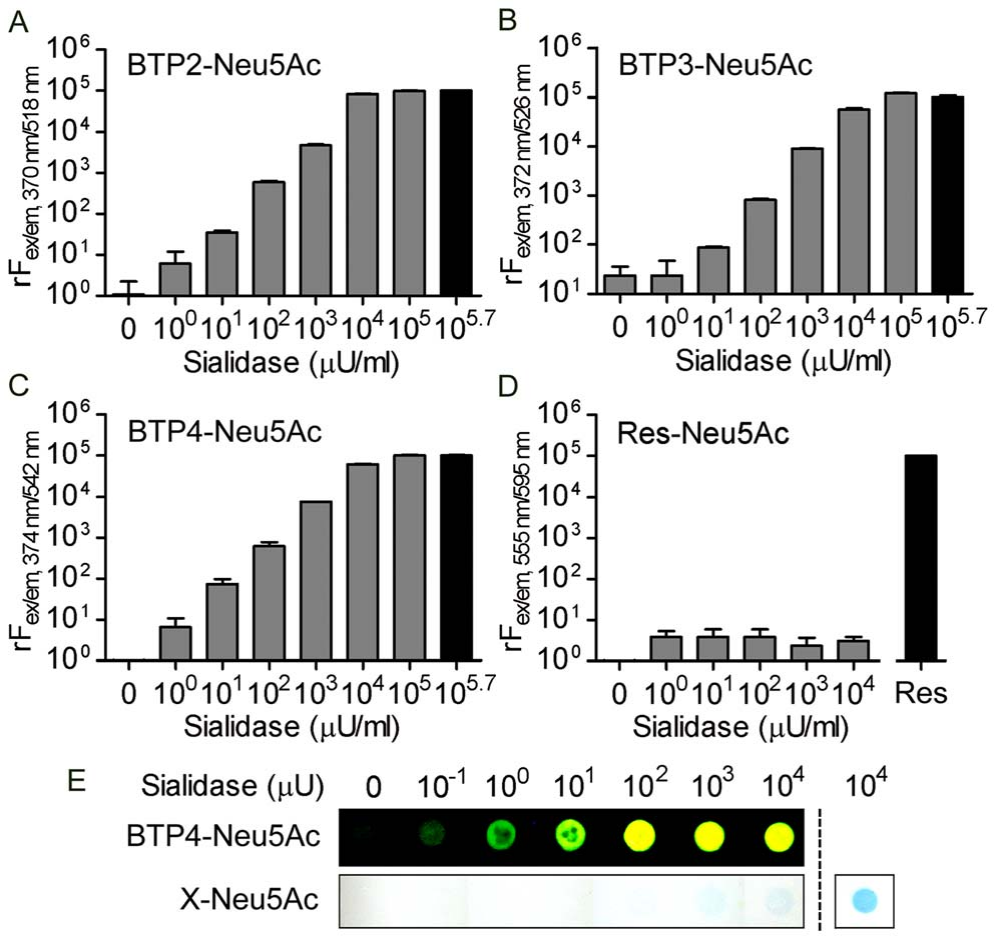
Hydrolysis of BTP-Neu5Ac with bacterial sialidase. A–D, Relative fluorescence intensities proportionally increased with increasing amounts of AUSA in 10 µM BTP2-Neu5Ac (A), BTP3-Neu5Ac (B) and BTP4-Neu5Ac (C) but not in 10 µM Res-Neu5Ac (D). Each bar and line represent the mean ± S.E.M. (n = 3). The fluorescence intensities of each black bar were set to 10^5^. Res: 10 µM resorufin. E, AUSA blotted on PVDF membranes was stained with 10 µM BTP4-Neu5Ac or X-Neu5Ac. The PVDF membranes were observed under UV light for BTP4-Neu5Ac or visible light for X-Neu5Ac. The blue color caused by staining of AUSA with 100 µM X-Neu5Ac is shown at the right of the dotted line.

We synthesized BTP-based sialic acid derivatives (BTP-Neu5Ac), BTP2-Neu5Ac, BTP3-Neu5Ac and BTP4-Neu5Ac, according to the synthetic scheme shown in Figure 2. These sialic acid derivatives were water-soluble and showed little fluorescence. When BTP2-Neu5Ac, BTP3-Neu5Ac and BTP4-Neu5Ac were incubated in ACSF containing 10^0^–10^5.7^ µU ml^−1^ sialidase from *Arthrobacter ureafaciens* (AUSA) at 37°C for 60 min (pH 7.3), fluorescent intensities were increased in proportion to the concentration of AUSA and reached a plateau at high concentrations (Figure 3 A–C). Extinction coefficients (M^−1^ cm^−1^) of BTP2, BTP3 and BTP4 in chloroform were 14970, 12440 and 14350, respectively. These results indicated that BTP2-Neu5Ac, BTP3-Neu5Ac and BTP4-Neu5Ac are useful for quantitative analysis of sialidase activities.

Resorufin is a water-insoluble fluorophore with a molecular size similar to that of BTP-Neu5Ac. Resorufin-based glycosidase substrates have been used for cytochemical staining and measurement of enzyme activity [16]. In the case of sialic acid derivatives with resorufin (Res-Neu5Ac), fluorescent intensities were not significantly changed by AUSA (one-way ANOVA, Figure 3 D). BTP-Neu5Ac would be more suitable than Res-Neu5Ac to fit the reaction pocket of the sialidase.

AUSA blotted on a PVDF membrane with different concentrations was stained with 10 µM BTP4-Neu5Ac. As a result of observation under UV light, fluorescent intensities could be changed in a wide dynamic range (Figure 3 E, upper). The sensitivity of BTP4-Neu5Ac was remarkably higher than that of X-Neu5Ac (Figure 3 E, bottom).

### Visualization of sialidase activity in rat acute brain slices

**Figure 4:**
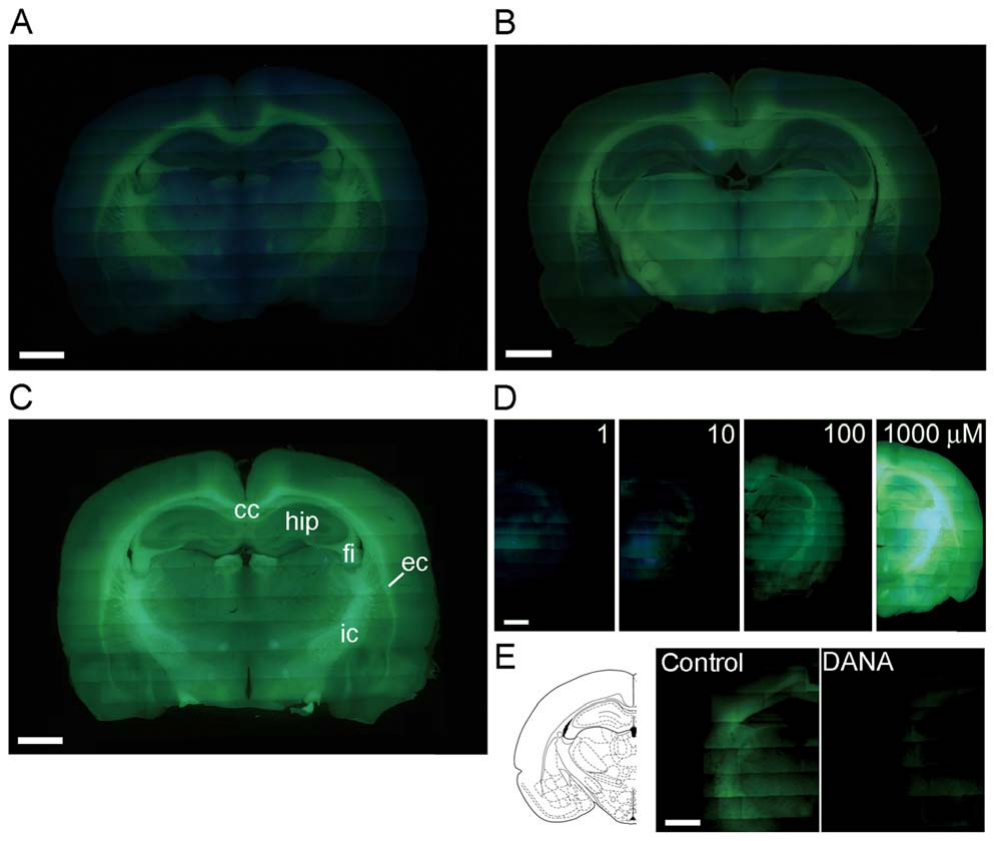
Visualizing of sialidase activities in rat brain slices with BTP-Neu5Ac. A–C, Sialidase activity was imaged in acute coronal slices of adult rat brains with BTP2-Neu5Ac (A), BTP3-Neu5Ac (B) and BTP4-Neu5Ac (C) at pH 7.3. Abbreviations: cc, corpus callosum; ec, external capsule; fi, fimbria; hip, hippocampus; ic, internal capsule. D, Brain slices were stained with various concentrations (1–1000 µM) of BTP4-Neu5Ac. E, Sialidase activity was imaged with 10 µM BTP4-Neu5Ac or 10 µM BTP4-Neu5Ac containing 1 mM DANA, a sialidase inhibitor. Emission filters that transmit above 420 and 510 nm were used in A–D and E, respectively. Scale bars in each panel represent 2 mm.

To visualize sialidase activity in the rat brain, acute brain slices were incubated with ACSF (pH 7.3) containing 1 mM BTP2-Neu5Ac, 1 mM BTP3-Neu5Ac or 1 mM BTP4-Neu5Ac at 27°C for 60 min. Incubation chambers were continuously bubbled with 95% O_2_ and 5% CO_2_ during staining to keep the slice condition healthy. After washing with ACSF, white matter showed intense fluorescence in the slices stained with BTP2-Neu5Ac, BTP3-Neu5Ac and BTP4-Neu5Ac (Figure 4 A–C). This result was consistent with results of the previous studies showing that white matter of the mammalian brain exhibits intense sialidase activity using 4MU-Neu5Ac or X-Neu5Ac with FRV LB [8], [17].

Since BTP4 is excited more efficiently with the excitation band pass filter (330–385 nm) of a fluorescence microscope than BTP2 or BTP3, imaging with BTP4-Neu5Ac exhibited the most intense fluorescence in these BTP-Neu5Ac series. We also analyzed the sensitivity and specificity of BTP4-Neu5Ac toward sialidase. In the case of sialidase activity imaging with X-Neu5Ac and FRV LB, a high concentration of X-Neu5Ac, generally 1 mM, is needed to improve sensitivity and specificity toward mammalian sialidase activity [7], [8]. On the other hand, BTP4-Neu5Ac can detect sialidase activities with low substrate concentrations even less than 10 µM (Figure 4 D). When 2,3-dehydro-2-deoxy-N-acetylneuraminic acid (DANA, 1 mM), a sialidase inhibitor, was applied during staining with BTP4-Neu5Ac, fluorescence was remarkably attenuated in the whole brain area (Figure 4 E). BTP-Neu5Ac is specifically hydrolyzed with sialidase, resulting in a remarkable increase in fluorescent intensity.

Since BTP-Neu5Ac, a hydrophilic substrate, filled in the extracellular space was hydrolyzed with mammalian sialidase of living tissues under physiological extracellular conditions, sialidase would cleave BTP-Neu5Ac mainly on the cell surface. The plasma membrane sialidase NEU3 hydrolyzed ganglioside efficiently and low-molecular-weight substrates such as 4MU-Neu5Ac slightly [18], [19]. Although the optimum pH of NEU3 is 3.8, NEU3 hydrolyzes ganglioside at even neutral pH [19], [20]. The lysosomal sialidase NEU1 was also reported to be present on plasma membranes and to hydrolyze 4MU-Neu5Ac efficiently [21], [22], [23]. Additionally, the cytosolic sialidase NEU2 has a membrane-bound form and hydrolyzes 4MU-Neu5Ac [24]. Therefore, not only NEU3 but also other sialidase isozymes would be involved in the sialidase activity detected with BTP-Neu5Ac.

### Cytotoxicity assay of BTP-Neu5Ac and BTP

**Figure 5:**
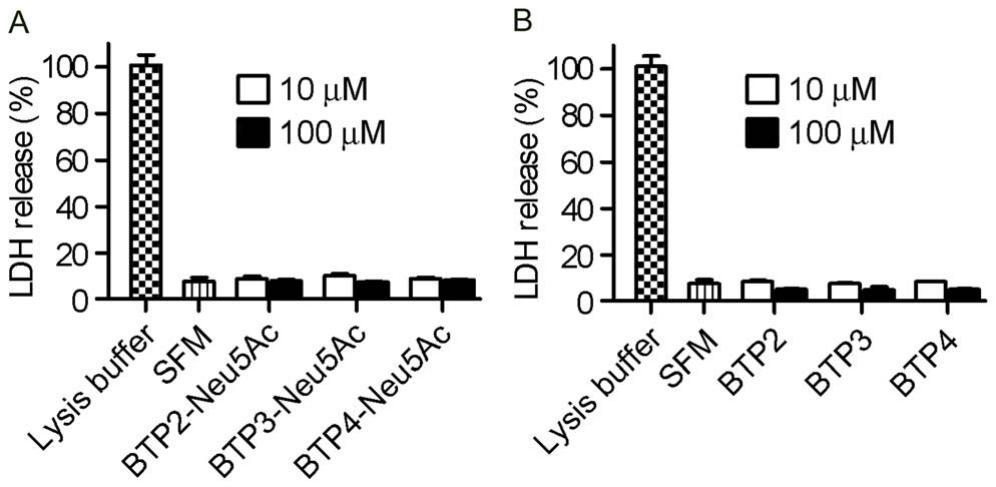
Undetectable cytotoxicity of BTP-Neu5Ac and BTP for 16-h exposure. MDCK cells were exposed to a serum-free medium (SFM) containing 10 or 100 µM BTP-Neu5Ac (A) or BTP (B) for 16 hr and released LDH was measured. LDH release is shown as relative to complete LDH release (100%) by treatment with lysis buffer.

For the cytotoxicity measurement, MDCK cells were exposed to BTP-Neu5Ac or BTP for 6 (data not shown) or 16 (Figure 5) hr. As a result of measurement for the amounts of lactate dehydrogenase (LDH) release from cells with damaged membrane, no significant cytotoxicity was detected for BTP-Neu5Ac and BTP (one-way ANOVA).

### Detection of cancer cells in mouse colon tissues

Recently, technology for cancer imaging has progressed remarkably. For example, Oku *et al.* developed a novel positron emitter-labeled liposome for positron emission tomography (PET) to image a small brain cancer noninvasively in real time [25]. Urano *et al.* developed a cancer-targeting monoclonal antibody conjugated with pH-activatable fluorescence probes for *in vivo* tumor detection [26]. A highly sensitive and specific fluorescent cancer probe has advantages for detecting small cancers because the minimum size of a tumor detected by using fluorescence endoscopy (approximately ∼1 mm) is much smaller than that by PET, computed tomography (CT) and magnetic resonance imaging (MRI) (approximately 5–20 mm) [27], [28]. Earlier detection of cancers and following rapid cure of them effectively prevents not only malignant alteration of cancers but also distal metastasis and strongly promises improvement of prognosis.

**Figure 6:**
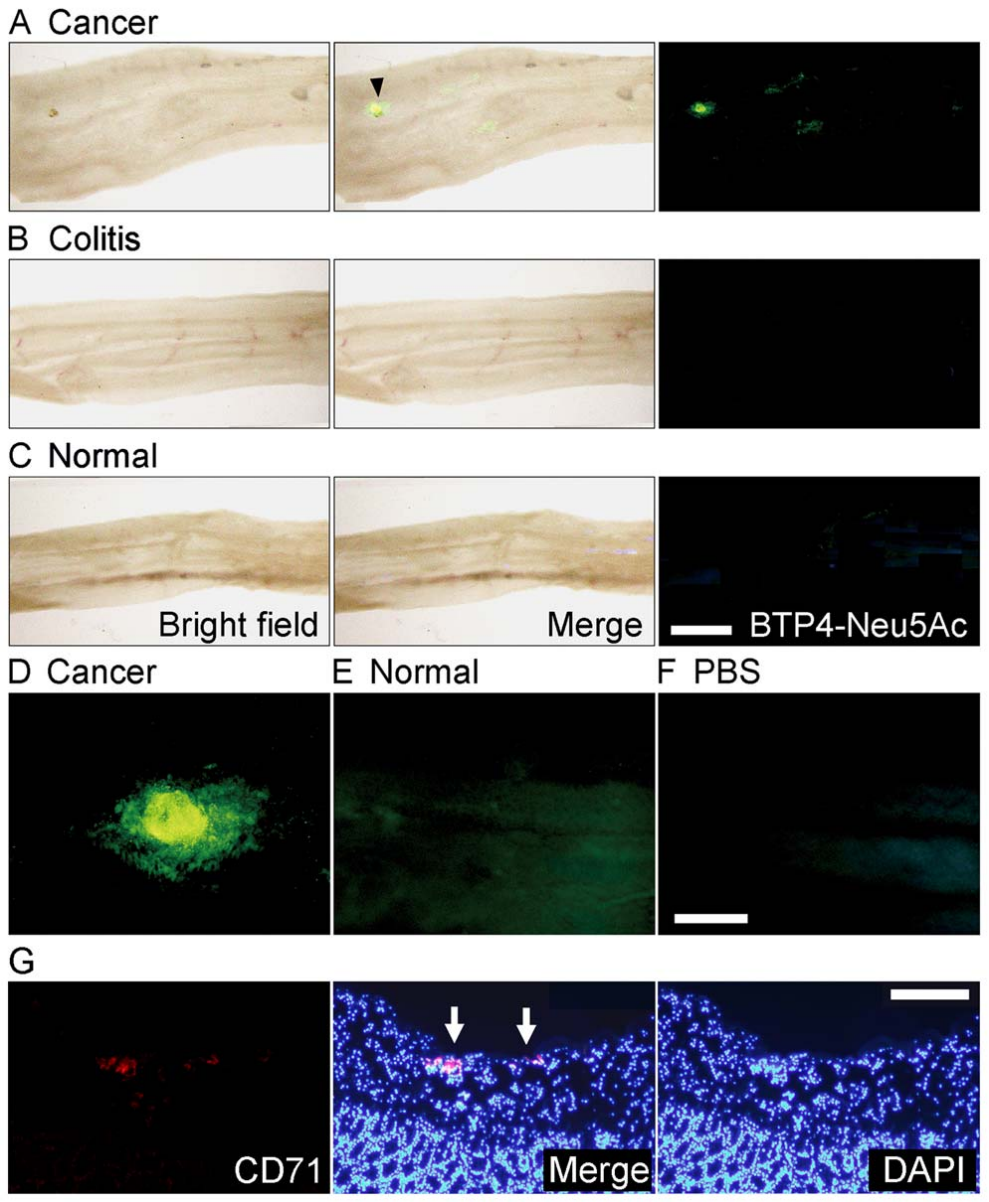
Detection of orthotopic colon cancer with BTP4-Neu5Ac in mouse colon tissue. A, One week after orthotopic colon implantation of Colon26 NL-17 cells, living colon tissues were stained with BTP4-Neu5Ac. Arrowhead indicates the cancer region. B and C, Inflammatory (B) or normal (C) colons were also stained with BTP4-Neu5Ac. Left, middle and right panels in panel A–C show bright field, merged and fluorescent views, respectively. D and E, Enlarged image of cancer (D) and normal (E) region stained with BTP4-Neu5Ac. F, Background fluorescence level of panels D and E is shown. G, Immunohistochemical staining (red fluorescence) by using rabbit anti-CD71 antibody and PE-conjugated goat anti-rabbit IgG antibody and nuclear staining with DAPI (blue fluorescence) were performed to detect colon cancer in cross sections of the mouse colon tissues that were used for sialidase activity imaging in panels A and D. Arrows indicate the regions showing intense fluorescence of BTP in panel A and D. Scale bar in panel C represents 2.5 mm and is common in panels A and B. Scale bar in panel F represents 0.5 mm and is common in panels D and E. Scale bar in panel G represents 0.2 mm.

Since colon cancer is reported to exhibit intense enzyme activity of a plasma membrane-associated sialidase [29], we tried to detect colon cancer with BTP4-Neu5Ac in living colon tissues. Mice were implanted orthotopically with Colon26 NL-17 cells, which are highly metastatic cancer cells isolated from a mouse colon adenocarcinoma 26. After 1 or 2 weeks, colon tissues were harvested and incubated in phosphate-buffered saline (PBS) containing 100 µM BTP4-Neu5Ac. Intense fluorescence was observed in the colon implanted with Colon26 NL-17 cells but not in inflammatory or normal colons (Figure 6 A–C, Figure S1). Whereas normal colon tissue showed weak fluorescence, fluorescence of colon cancer was much stronger than that of normal tissue (Figure 6 D–F). The regions showing intense fluorescence in the colon implanted with Colon26 NL-17 cells were confirmed to possess cancer by immunohistochemical staining (Figure 6 G). Therefore, colon cancers could be distinguished easily from normal tissues by using BTP-Neu5Ac.

Sialidase activities expressed in Colon26 NL-17 were reported to be weakest among colon adenocarcinoma 26 sublines such as NL-4, NL-17, NL-22 and NL-44 [30]. Since even Colon 26 NL-17 adenocarcinoma implanted orthotopically in mouse colons were detected with BTP-Neu5Ac clearly, BTP4-Neu5Ac would be sensitive enough for cancer probe.

### Detection of cancer in human colon tissues

**Figure 7:**
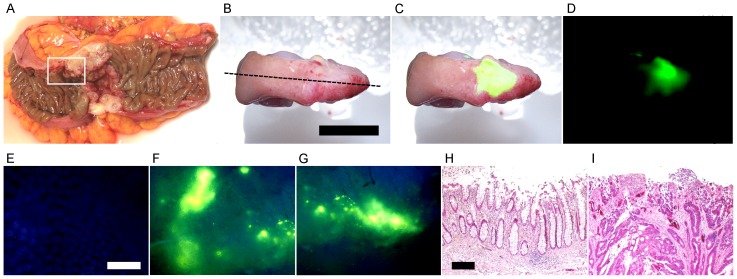
Detection of human colon cancer with BTP4-Neu5Ac. A, A human colon cancer specimen (region enclosed with a white line) was obtained from surgical cancer tissue (UICC T classification: T3). B–D, The colon tissue was stained with BTP4-Neu5Ac. B, C and D show photographic, merged and fluorescent images, respectively. E–G, Enlarged fluorescence images of normal (E) and cancer (F and G) regions were acquired with a fluorescent microscope. H and I, A longitudinal slice of colon tissue was prepared at the dotted line in panel B. Non-fluorescence and fluorescence regions in panel C were stained with hematoxylin-eosin and are shown in panels H and I, respectively. Scale bars in panel B, E and H represent 10 mm (common in panels B–D), 500 µm (common in panels E–G) and 250 µm (common in panels H and I), respectively.

It has been reported that the expression level of Neu3 mRNA in human colon cancer is 3–100-times higher than that in normal tissue [29]. Therefore, we also tried to detect human colon cancer with BTP4-Neu5Ac. After incubation of human colon cancer living tissues, categorized as T3 by the T classification of the Union for International Cancer Control (UICC), in PBS containing 200 µM BTP4-Neu5Ac, intense fluorescence was observed in the tumor regions but not in the normal regions (Figure 7 A–G). The regions showing intense fluorescence and non-fluorescence were confirmed to be cancer and normal tissues, respectively, by using hematoxylin-eosin staining (Figure 7 H and I). Cancer categorized as T4 was also detected with BTP4-Neu5Ac in human colon tissues (data not shown). Although additional evaluation of toxicity is needed, it would be possible to use BTP4-Neu5Ac for cancer detection not only in pathological examination but also noninvasive diagnosis.

## Conclusions

We developed novel fluorescent sialidase substrates for histochemical staining of sialidase activities in mammalian tissues. Since sialidase plays crucial roles in many biological functions including lysosomal catabolism, immune system and neural functions [2], [31], BTP-Neu5Ac is a powerful tool for detailed investigation of sialidase functions. Since the cytotoxicities of BTP-Neu5Ac and BTP were undetectable, BTP-Neu5Ac can be used for biological assay of sialidase in the living tissue, e.g. time-lapse imaging. Finally, colon cancers were detected with BTP4-Neu5Ac in a living tissue, suggesting that BTP-Neu5Ac would be also useful for cancer probes.

## Supporting Information

Figure S1 **Detection of colon cancer with BTP4-Neu5Ac in two weeks after implantation of cancer cells.** Two weeks after orthotopical implantation of Colon26 NL-17 cells, mouse colons were stained with BTP4-Neu5Ac. Inflammatory or normal colons were also stained with BTP4-Neu5Ac. Left and right panels show fluorescent images and bright field images merged with fluorescent images, respectively. Scale bar represents 5.0 mm.
(TIF)

File S1 **Synthetic procedures and ^1^H spectra of new compounds.**
(PDF)
