# Supplementary figures and images for: Visualization of Sialidase Activity in Mammalian Tissues and Cancer Detection with a Novel Fluorescent Sialidase Substrate
Source: PLoS One. 2014 Jan 10;9(1):e81941. doi: 10.1371/journal.pone.0081941 (PMC3888388; doi:10.1371/journal.pone.0081941)

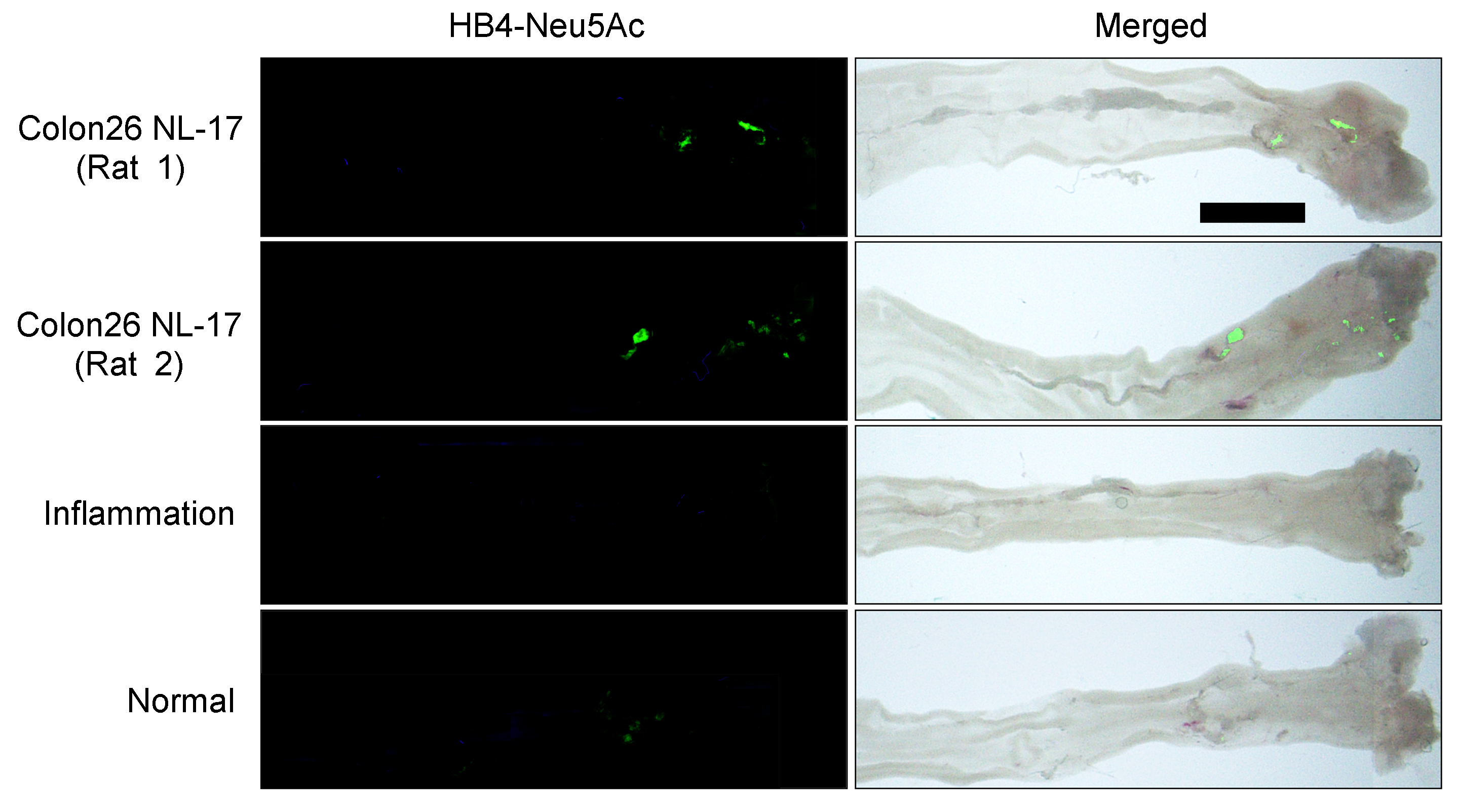

Supplement: Figure S1 — Detection of colon cancer with BTP4-Neu5Ac in two weeks after implantation of cancer cells. Two weeks after orthotopical implantation of Colon26 NL-17 cells, mouse colons were stained with BTP4-Neu5Ac. Inflammatory or normal colons were also stained with BTP4-Neu5Ac. Left and right panels show fluorescent images and bright field images merged with fluorescent images, respectively. Scale bar represents 5.0 mm. (TIF) [file pone.0081941.s001.tif]
