# Supplementary material for: Visualization of Sialidase Activity in Mammalian Tissues and Cancer Detection with a Novel Fluorescent Sialidase Substrate
Source: PLoS One. 2014 Jan 10;9(1):e81941. doi: 10.1371/journal.pone.0081941 (PMC3888388; doi:10.1371/journal.pone.0081941)
Supplement: File S1 — Synthetic procedures and 1H spectra of new compounds. (PDF) [file pone.0081941.s002.pdf]

1. Synthetic procedures

1-1 Materials

All chemicals used in the syntheses were purchased from Tokyo Chemical Industry Co., Ltd., Wako Pure Chemical Industries, Ltd., or Sigma-Aldrich Co. LLC. and used without further purification.

1-2 Methods for analysis of synthesized compounds

^{1}H-NMR experiments were carried out on a JMTC-600 (JEOL Ltd.) 600 NMR spectrometer with $CDCl_3$ or D_2O as a solvent. Chemical shifts are expressed in parts per million (ppm, δ) relative to the residual deuterated solvent or the internal standard tetramethylsilane. High-resolution mass spectra (HRMS) were measured on an AccuTOF (JMS-T100LC) equipped with an electrospray ion source (JEOL Ltd.).

1-3 Syntheses of compounds

1-3-1 General procedure for synthesis of BTP2, BTP3 and BTP4

The procesure for synthesis procedure of (2-benzothiazol-2-yl)-4-bromophenol (BTP3) is described in the following as a typical procedure for synthesis of 2-benzothiazol-2-yl phenol derivatives (BTP2, BTP3 and BTP4).[S1] According to the modified procedure previously reported,[S2] to a solution of 5-bromosalicylaldehyde (18.1 g, 90 mmol) in THF (25 mL) was added a solution of 2-aminothiophenol (11.3 g, 90 mmol) in EtOH (75 mL) and stirred at 80 °C for 28 hours. After cooling to room temperature, the solvent was removed under reduced pressure, and the resulting precipitate was suspended in Et_2O, filtered, washed with Et_2O, and dried to give 13.75 g of desired product BTP3 in 50% yield as pale yellow green needles. The mother liquor

was concentrated, and the obtained precipitate was filtered again to give a second crop (1.95 g, 7.1% yield) as pale yellow needles.

(2-benzothiazol-2-yl)-phenol (BTP2) [S3]: 67% yield.

(2-benzothiazol-2-yl)-4,6-dibromophenol (BTP4) [S1]: 57% yield.

1-3-2 General procedure for synthesis of 1a, 1b and 1c

Synthesis procedure of **1b** is described in the following as typical synthesis procedure of methyl 2-(benzothiazol-2-yl)phenyl (5-acetamido-4,7,8,9-tetra-O-acetyl-3,5-dideoxy-D-glycero-α-D-galacto-2-nonulopyranosid)onate derivatives (**1a**, **1b** and **1c**). To a solution of methyl D-*N*-acetylneuraminate [S4] (1.0 g, 3.09 mmol) in AcOH (10 ml) was added acetyl chloride (10 ml), and the solution was sealed and allowed to stand at room temperature for 18 hours. The solvent was removed under reduced pressure and dried azeoptropically with toluene three times to give methyl 4,7,8,9-tetra-*O*-acetyl-2-chloro-D-*N*-acetylneuraminate as a colorless amorphous. The residue was used for the following reaction without further purification. To a suspension of NaH (60% in mineral oil, 136 mg, 3.4 mmol) in THF (10 mL) was slowly added a solution of 2-(benzothiazol-2-yl)-4-bromophenol (947 mg, 3.09 mmol) in DMF (10 mL) with water cooling, stirred for 10 minutes. To the reaction mixture was added a solution of the crude chloride in THF (10 ml) over a period of a few minutes, and stirred at room temperature for 19 hours. The reaction mixture was diluted with AcOEt, and the organic layer was washed with water (3 times) and brine, dried over Na_2SO_4, and concentrated under reduced pressure. The residue was purified by silica gel column chromatography (silica gel: 150 mL, AcOEt as an eluent) to give 1.74 g of

desired product **1b** in 72% yield in two steps as a colorless amorphous after freeze-drying from dioxane.

Methyl 2-(benzothiazol-2-yl)-4-bromophenyl-(5-acetamido-4,7,8,9-tetra-*O*-acetyl-3,5-dideoxy -D-glycero-α-D-galacto-2-nonulopyranosid)onate (**1b**):

^{1}H-NMR (600MHZ, $CDCl_3$) δ 8.62 (d, *J* = 2.4 Hz, 1H), 8.11 (d, *J* = 7.8 Hz, 1H), 7.96 (d, *J* = 8.4 Hz, 1H), 7.56-7.52 (m, 2H), 7.43 (dd, *J* = 7.8 Hz, 1H), 7.29-7.26 (m, 1H), 5.44 (ddd, *J* = 3.0, 4.8, 9.0 Hz, 1H), 5.40 (dd, *J* = 1.8, 9.0 Hz, 1H), 5.22 (d, *J* = 9.6 Hz, 1H), 5.00 (ddd, *J* = 4.8, 12.6, 10.2 Hz, 1H), 4.62 (dd, *J* = 1.8, 10.8 Hz, 1H), 4.28 (dd, *J* = 3.0, 13.2 Hz, 1H), 4.20 (ddd, *J* = 9.6, 10.2 Hz, 1H), 4.15 (dd, *J* = 4.8, 13.2 Hz, 1H), 3.56 (s, 3H), 3.00 (dd, *J* = 4.8, 12.6 Hz, 1H), 2.62 (t, *J* = 12.6 Hz, 1H), 1.95, 2.08, 2.10, 2.13, 2.21 (s, 15H).

HR-MS (TOF-MS) Calcd for $C_{33}H_{35}Br\ N_2NaO_{13}S$ $[M+Na]^+$: 801.09409; Found: 801.09165.

Methyl 2-(benzothiazol-2-yl)phenyl-(5-acetamido-4,7,8,9-tetra-*O*-acetyl-3,5-dideoxy-D-glycero -α-D-galacto-2-nonulopyranosid)onate (**1a**):

37% yield in two steps. ^{1}H-NMR (600MHZ, $CDCl_3$) δ 8.45 (dd, *J* = 1.8, 7.8 Hz, 1H), 8.10 (d, *J* = 8.4 Hz, 1H), 7.96 (d, *J* = 7.8 Hz, 1H) ,7.52-7.50 (m, 1H), 7.45-7.37 (m, 3H), 7.23 (d, *J* = 8.4 Hz, 1H), 5.45 (ddd, *J* = 3.0, 4.8, 8.4 Hz, 1H), 5.41 (dd, *J* = 1.8, 8.4 Hz, 1H), 5.21 (d, *J* = 9.6 Hz, 1H), 5.01 (ddd, *J* = 4.8, 10.2, 13.2 Hz, 1H), 4.61 (dd, *J* = 1.8, 10.2 Hz, 1H), 4.31 (dd, *J* = 3.0, 12.0 Hz, 1H), 4.19 (ddd, *J* = 9.6, 10.2 Hz, 1H), 4.17 (dd, *J* = 4.8, 12.0 Hz, 1H), 3.53 (s, 3H), 3.00 (dd, *J* = 4.8, 13.2 Hz, 1H), 2.62 (t, *J* = 13.2 Hz, 1H), 1.94, 2.07, 2.09, 2.12, 2.20 (s, 15H).

HR-MS (TOF-MS) Calcd for $C_{33}H_{37}N_2O_{13}S$ $[M+Na]^+$: 701.20163; Found: 701.20118.

Methyl 2-(benzothiazol-2-yl)-4,6-dibromophenyl-(5-acetamido-4,7,8,9-tetra-*O*-acetyl-3,5- dideoxy -D-glycero-α-D-galacto-2-nonulopyranosid)onate (**1c**):

37% yield in two steps. ^{1}H-NMR (600MHZ, $CDCl_3$) δ 8.26 (d, *J* = 1.8 Hz, 1H), 8.11 (d, *J* = 8.4 Hz, 1H), 7.99 (d, *J* = 7.8 Hz, 1H), 7.79 (d, *J* = 2.4 Hz, 1H), 7.52-7.50 (m, 1H), 7.42-7.40 (m, 1H), 5.03-5.07 (m, 1H), 5.01 (dd, *J* =1.8, 8.4 Hz, 1H), 4.75-4.73 (m, 1H), 3.94 (dd, *J* = 1.8, 13.2 Hz, 1H), 3.71 (s, 3H), 3.64 (dd, *J* = 4.2, 13.2 Hz, 1H), 3.49 (dd, *J* = 1.8, 10.8 Hz, 1H), 3.06 (dd, *J* = 4.8, 13.2 Hz, 1H), 2.26 (t, *J* = 13.2 Hz, 1H), 1.80, 1.94, 1.99, 2.04, 3.68 (s, 15H).

HR-MS (TOF-MS) Calcd for $C_{33}H_{34}Br_2$ $N_2NaO_{13}S$ $[M+Na]^+$: 879.00460; Found: 879.00357.

Note: The yield of **1b** was higher than the yield of **1a** and **1c**. Since the amounts of the starting material used (methyl D-*N*-acetylneuraminate) were 1.0 g for the synthesis of **1b** but 50 - 100 mg for the synthesis of **1a** and **1c**, each step progressed with minimal loss in the synthesis of **1b**.

1-3-3 General procedure for synthesis of BTP2-Neu5Ac, BTP3-Neu5Ac and BTP4-Neu5Ac

Synthesis procedure of BTP3-Neu5Ac is described in the following as typical synthesis procedure of 2-(benzothiazol-2-yl)phenyl-5-acetamido-3,5-dideoxy-α-D-glycero-D-galacto-2-nonulopyranosidonic acid derivatives (BTP2-Neu5Ac, BTP3-Neu5Ac and BTP4-Neu5Ac). To a solution of **1b** (144 mg, 185 μmol) in MeOH (3 ml) at 0 ºC was added sodium methoxide solution (ca. 5 mol L^{-1} in MeOH, 100 μL) and stirred at room temperature for 6 hours. The reaction mixture was neutralized by addition of Amberlite IR-50(H^+), filtered, and concentrated under reduced pressure. The residue was dissolved in MeOH (2 mL), added to aqueous NaOH

solution (0.1 mol L^{-1}, 2 mL) at 0 ºC, and stirred at room temperature for 2 days. The reaction mixture was concentrated under reduced pressure. The residue was applied to Bio-Gel P2 (H_2O as an eluent) to give 56 mg of desired product BTP3-Neu5Ac in 51% yield in two steps as a colorless amorphous after freeze-drying from water.

<u>2-(Benzothiazol-2-yl)-4-bromophenyl-5-acetamido-3,5-dideoxy-α-D-glycero-D-galacto-2-nonulopyranosidonic acid (BTP3-Neu5Ac):</u>

^{1}H-NMR (600MHZ, D_2O) δ 8.02-8.01 (m, 1H), 7.85-7.82 (m, 2H), 7.41-7.36 (m,2H), 7.31-7.23 (m, 3H), 7.20-7.18 (m, 1H), 3.78 (dd, *J* = 10.2 Hz, 1H), 3.75 (ddd, *J* = 10.2 Hz, 1H), 3.63-3.58 (m, 3H), 3.45 (dd, *J* = 12.6, 6.0 Hz, 1H), 3.41 (d, *J* = 9.0 Hz, 1H), 2.80 (dd, *J* = 12.6, 4.8 Hz, 1H), 1.97 (dd, *J* = 12.6 Hz, 1H), 1.86 (s, 3H).

HR-MS (TOF-MS) Calcd for $C_{24}H_{24}Br N_2O_9S$ $[M-H]^-$: 595.03859; Found: 595.03953.

<u>2-(Benzothiazol-2-yl)phenyl-5-acetamido-3,5-dideoxy-α-D-glycero-D-galacto-2-nonulopyranosidonic acid (BTP2-Neu5Ac):</u>

49% yield in two steps. ^{1}H-NMR (600MHZ, D_2O) δ 7.97 (d, *J* = 8.4 Hz, 1H), 7.90-7.85 (m, 2H), 7.43-7.40 (m, 1H), 7.37-7.31 (m, 3H), 7.16-7.13 (m, 1H), 3.80 (dd, *J* = 10.2, 1.2 Hz, 1H), 3.73 (ddd, *J* = 9.6 Hz, 1H), 3.64-3.59 (m, 3H), 3.43 (dd, *J* = 12.6, 6.6 Hz, 1H), 3.40 (dd, *J* = 8.4, 1.2 Hz, 1H), 2.83 (dd, *J* = 12.0, 4.8 Hz, 1H), 1.86 (dd, *J* = 12.6 Hz, 1H), 1.86 (s, 3H).

HR-MS (TOF-MS) Calcd for $C_{24}H_{25}N_2O_9S$ $[M-H]^-$: 517.12808; Found: 517.12877.

<u>2-(Benzothiazol-2-yl)-4,6-dibromophenyl-5-acetamido-3,5-dideoxy-α-D-glycero-D-galacto-2-</u>

nonulopyranosidonic acid (BTP4-Neu5Ac):

38% yield in two steps. ^{1}H-NMR (600MHZ, D_2O) δ 7.88 (s, 1H), 7.72-7.70 (m, 2H), 7.34-7.32 (m, 1H), 7.21-7.16 (m, 2H), 3.79-3.75 (m, 2H), 3.69-3.66 (m, 2H), 3.63-3.58 (m, 3H), 3.48 (dd, *J* = 12.6, 6.0 Hz, 1H), 3.43 (d, *J* = 9.0 Hz, 1H), 2.81 (dd, *J* = 12.6, 4.8 Hz, 1H), 1.99 (dd, *J* = 12.6 Hz, 1H), 1.87 (s, 3H).

MS (TOF-MS) m/z: 676 $[M-H]^-$.

Note: The yield of BTP4-Neu5Ac was lower than the yields of BTP2-Neu5Ac and BTP3-Neu5Ac since the bulkiness of bromines could influence the demethylation efficiency of **1c**.

1-4 Supplementary Reference

(S1) Deligeorgiev, T. G., Kaloyanova, S., Vasilev, A., and Vaquero, J. J. (2010) Novel Green Procedure for the Synthesis of 2-Arylbenzothiazoles Under Microwave Irradiation in Peg 200 Or Peg 400, *Phosphorus Sulfur and Silicon and the Related Elements 185*, 2292-2302.

(S2) Mashraqui, S. H., Kumar, S., and Vashi, D. (2004) Synthesis, cation-binding and optical spectral studies of photoemmitive benzothiazole crown ethers, *Journal of Inclusion Phenomena and Macrocyclic Chemistry 48*, 125-130.

(S3) Azarifar, D., Maleki, B., and Setayeshnazar, M. (2009) A Simple, Microwave-Assisted, and Solvent-Free Synthesis of 2-Arylbenzothiazoles by Acetic Acid-Promoted Condensation of

Aldehydes with 2-Aminothiophenol in Air, *Phosphorus Sulfur and Silicon and the Related Elements 184*, 2097-2102.

(S4) Ogura, H., Furuhata, K., Itoh, M., and Shitori, Y. (1986) Syntheses of 2-O-Glycosyl Derivatives of N-Acetyl-D-Neuraminic Acid .3. - Studies on Sialic Acids, *Carbohydr. Res. 158*, 37-51.

2. ^{1}H spectra of New Compounds

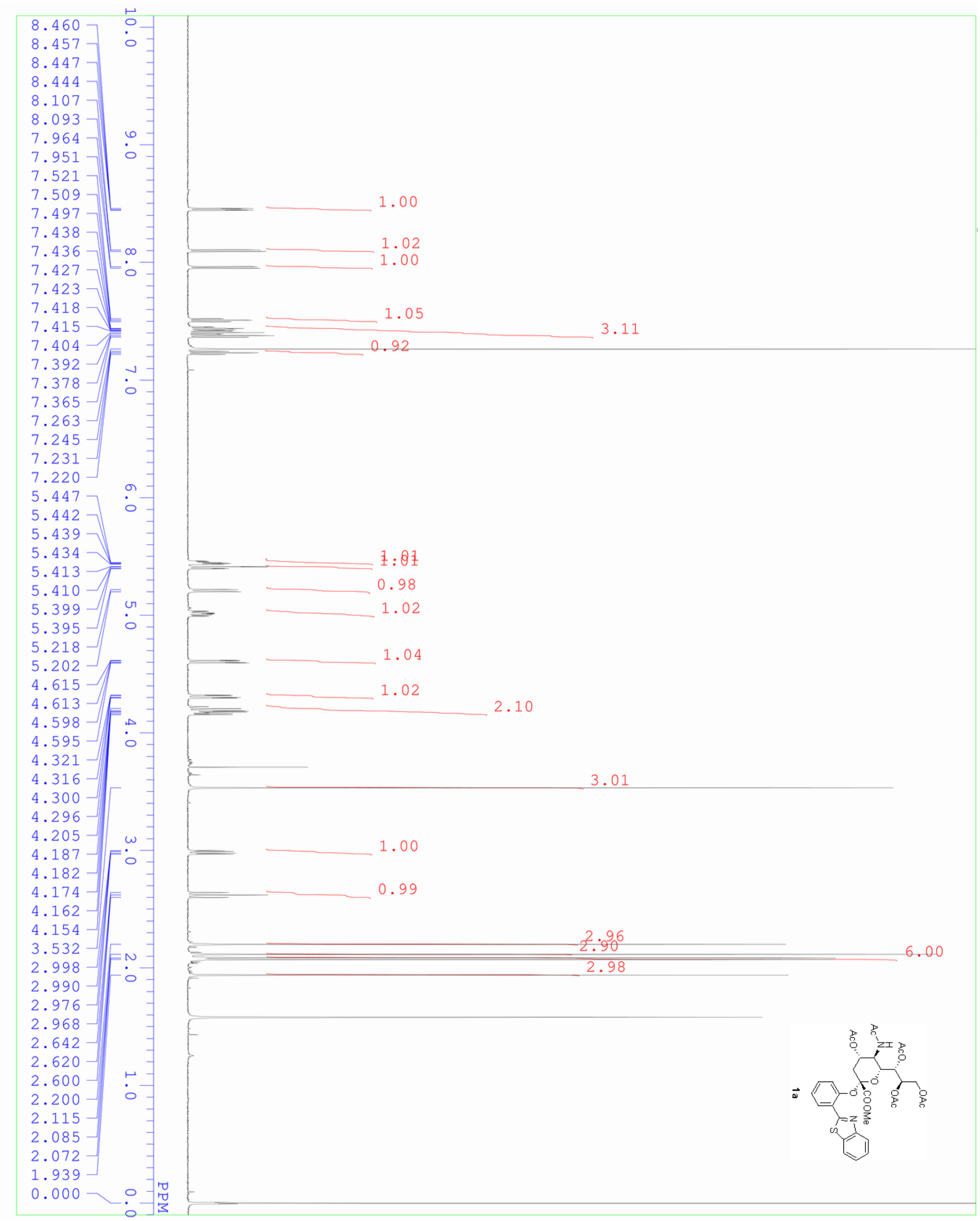

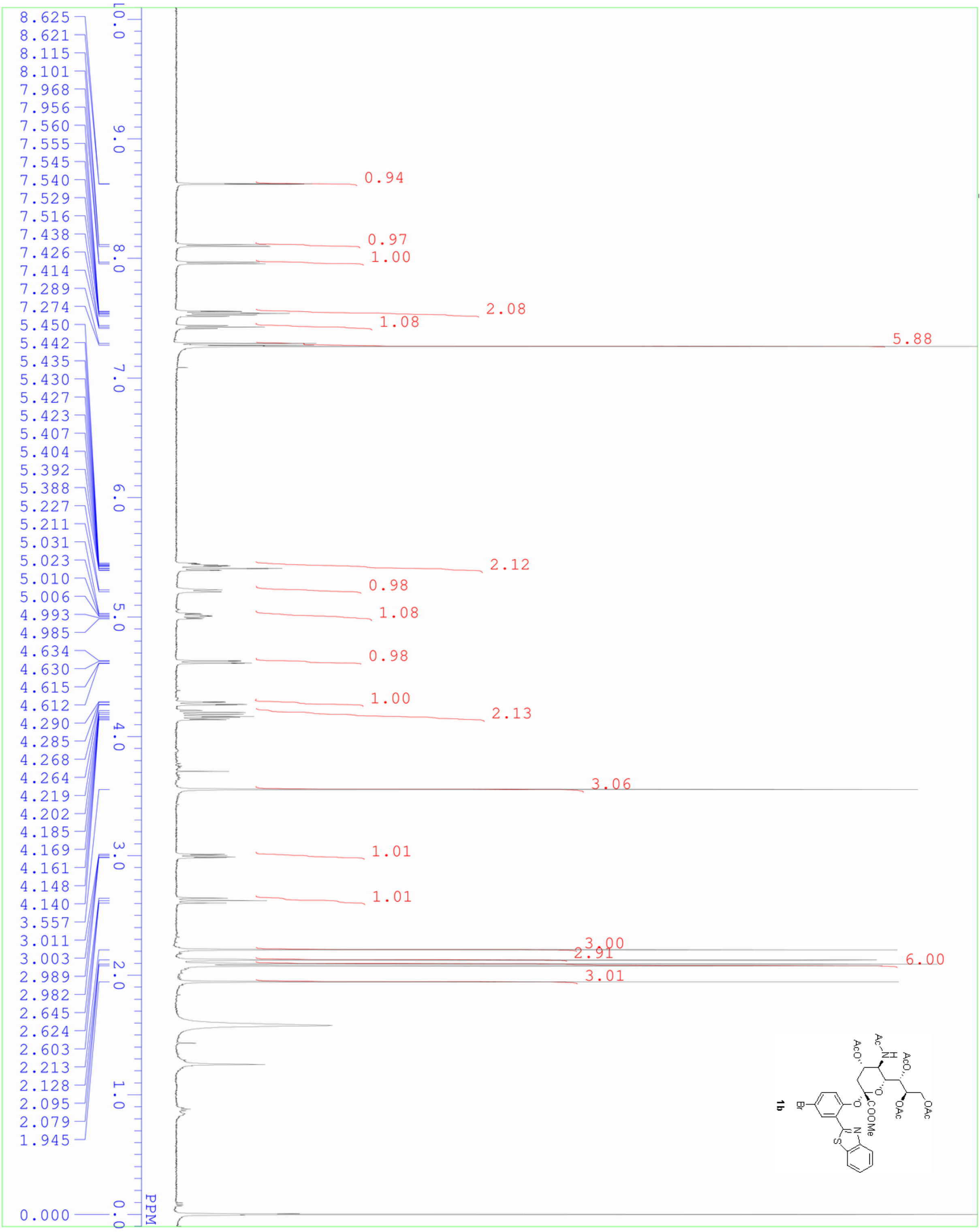
8.625
8.621
8.115
8.101
7.968
7.956
7.560
7.555
7.545
7.540
7.529
7.516
7.438
7.426
7.414
7.289
7.274
5.450
5.442
5.435
5.430
5.427
5.423
5.407
5.404
5.392
5.388
5.227
5.211
5.031
5.023
5.010
5.006
4.993
4.985
4.634
4.630
4.615
4.612
4.290
4.285
4.268
4.264
4.219
4.202
4.185
4.169
4.161
4.148
4.140
3.557
3.011
3.003
2.989
2.982
2.645
2.624
2.603
2.213
2.128
2.095
2.079
1.945
0.000
PPM
0.94
0.97
1.00
2.08
1.08
5.88
2.12
0.98
1.08
0.98
1.00
2.13
3.06
1.01
1.01
3.00
2.91
6.00
3.01
1b

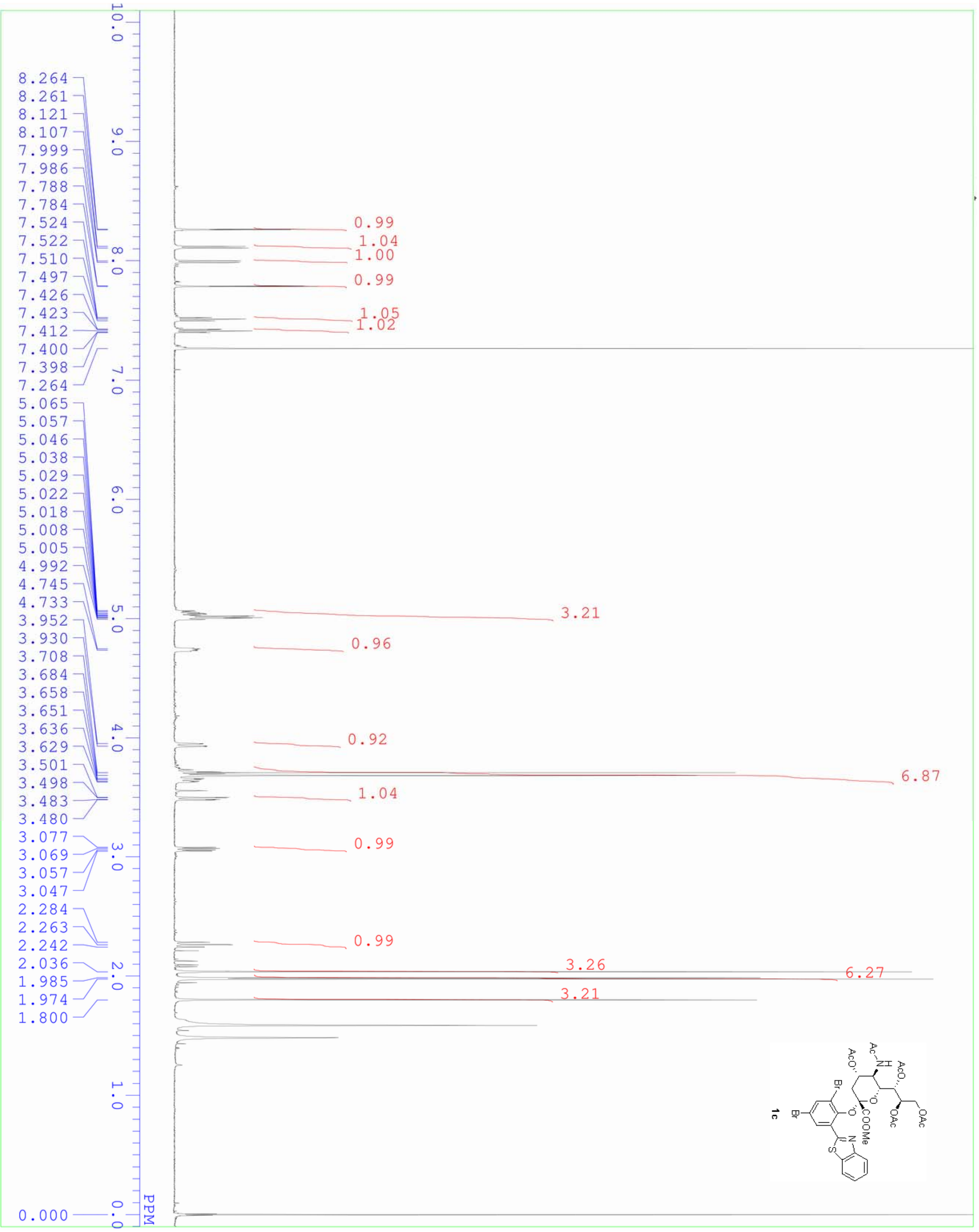
8.264
8.261
8.121
8.107
7.999
7.986
7.788
7.784
7.524
7.522
7.510
7.497
7.426
7.423
7.412
7.400
7.398
7.264
5.065
5.057
5.046
5.038
5.029
5.022
5.018
5.008
5.005
4.992
4.745
4.733
3.952
3.930
3.708
3.684
3.658
3.651
3.636
3.629
3.501
3.498
3.483
3.480
3.077
3.069
3.057
3.047
2.284
2.263
2.242
2.036
1.985
1.974
1.800
0.000
PPM
0.99
1.04
1.00
0.99
1.05
1.02
3.21
0.96
0.92
6.87
1.04
0.99
0.99
3.26
6.27
3.21
1c

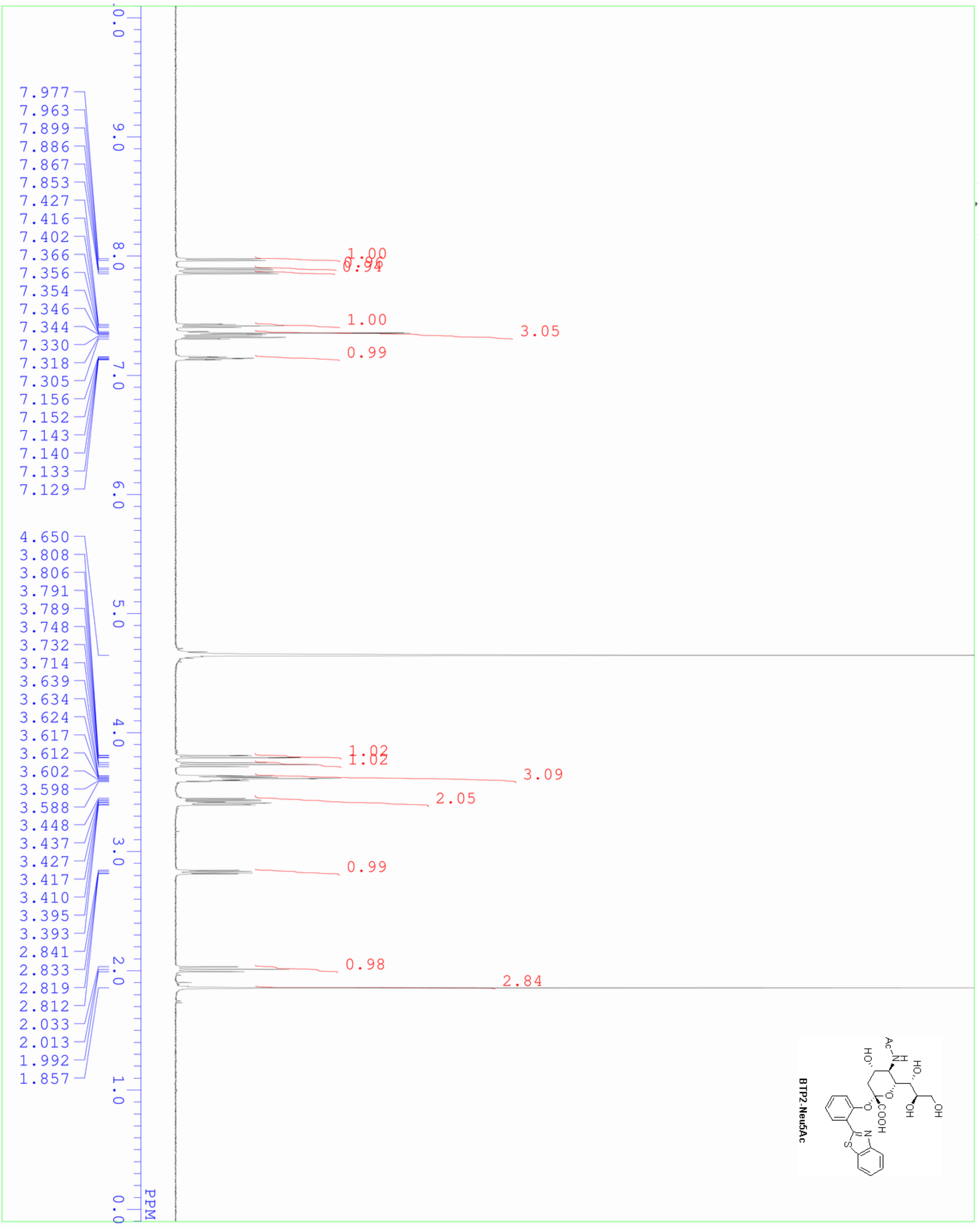
7.977
7.963
7.899
7.886
7.867
7.853
7.427
7.416
7.402
7.366
7.356
7.354
7.346
7.344
7.330
7.318
7.305
7.156
7.152
7.143
7.140
7.133
7.129
4.650
3.808
3.806
3.791
3.789
3.748
3.732
3.714
3.639
3.634
3.624
3.617
3.612
3.602
3.598
3.588
3.448
3.437
3.427
3.417
3.410
3.395
3.393
2.841
2.833
2.819
2.812
2.033
2.013
1.992
1.857
1.00
0.96
0.94
1.00
3.05
0.99
1.02
1.02
3.09
2.05
0.99
0.98
2.84
PPM
BTP2-Neu5Ac

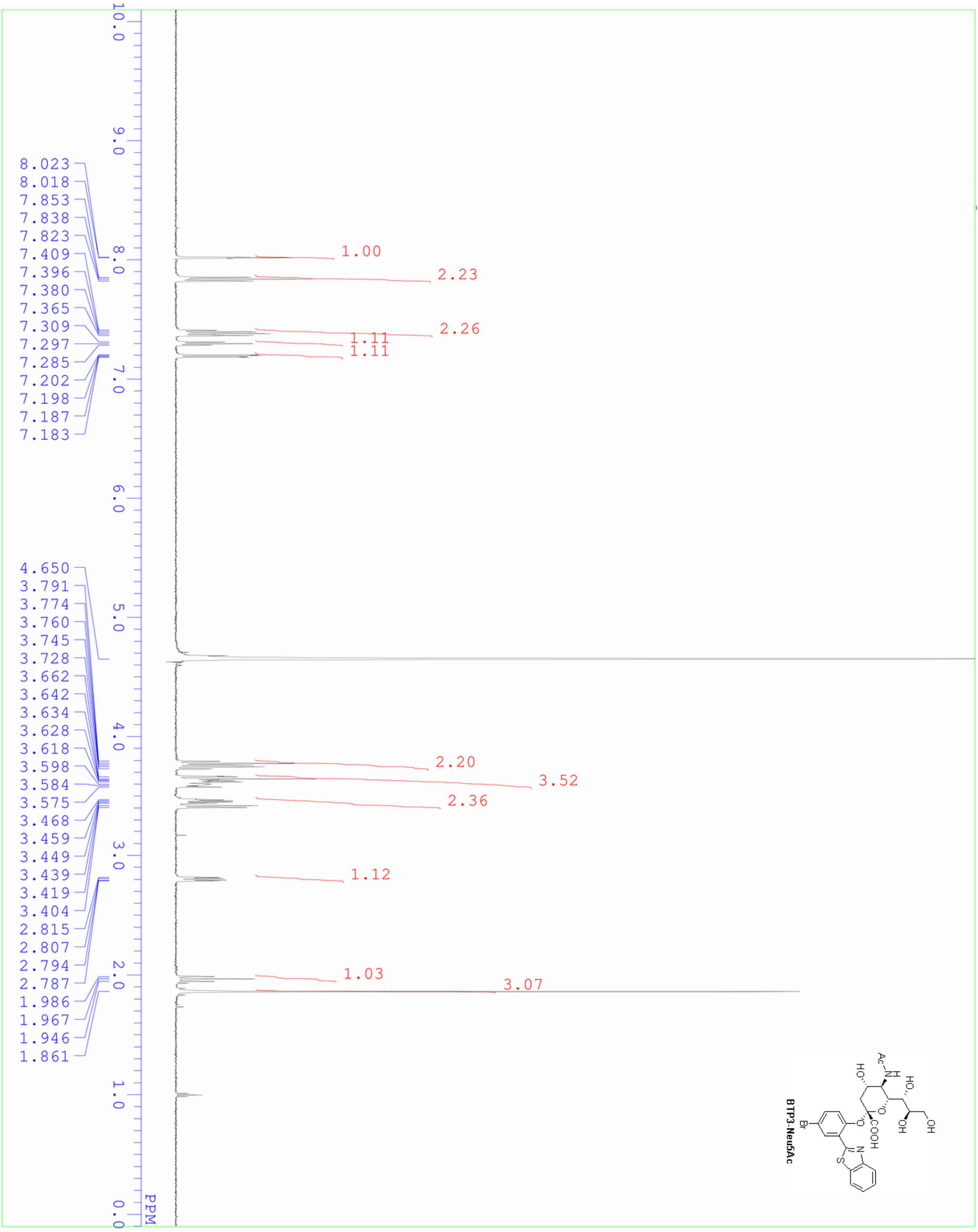
8.023
8.018
7.853
7.838
7.823
7.409
7.396
7.380
7.365
7.309
7.297
7.285
7.202
7.198
7.187
7.183
4.650
3.791
3.774
3.760
3.745
3.728
3.662
3.642
3.634
3.628
3.618
3.598
3.584
3.575
3.468
3.459
3.449
3.439
3.419
3.404
2.815
2.807
2.794
2.787
1.986
1.967
1.946
1.861
1.00
2.23
2.26
1.11
1.11
2.20
3.52
2.36
1.12
1.03
3.07
PPM
BTP3-Neu5Ac

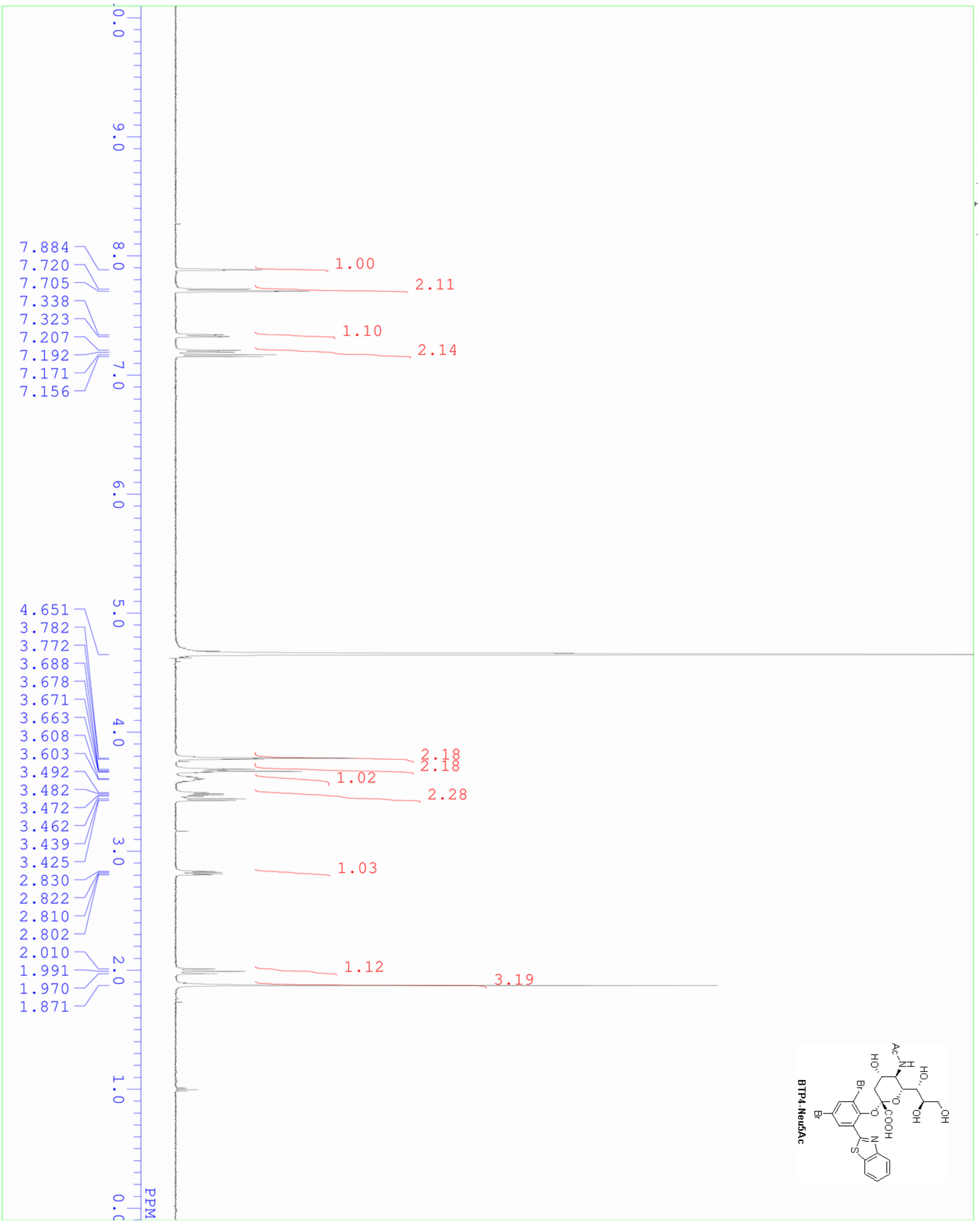
7.884
7.720
7.705
7.338
7.323
7.207
7.192
7.171
7.156
4.651
3.782
3.772
3.688
3.678
3.671
3.663
3.608
3.603
3.492
3.482
3.472
3.462
3.439
3.425
2.830
2.822
2.810
2.802
2.010
1.991
1.970
1.871
1.00
2.11
1.10
2.14
2.18
2.18
1.02
2.28
1.03
1.12
3.19
PPM
BTP4-Neu5Ac
